# Prediction and analysis of trends in the nutritional status of children under 5 years in Iran: reanalysis of the results of national surveys conducted between 1998 and 2020

**DOI:** 10.3389/fnut.2023.1083318

**Published:** 2023-05-12

**Authors:** Delaram Ghodsi, Hamid Rasekhi, Zahra Yari, Roshanak Roustaee, Bahereh Nikooyeh, Ayoub Faramarzi, Hassan Eini-Zinab, Tirang R. Neyestani

**Affiliations:** ^1^Department of Nutrition Research, National Nutrition and Food Technology Research Institute, Faculty of Nutrition Sciences and Food Technology, Shahid Beheshti University of Medical Sciences, Tehran, Iran; ^2^Department of Community Nutrition, Faculty of Nutrition Sciences and Food Technology, National Nutrition and Food Technology Research Institute, Shahid Beheshti University of Medical Sciences, Tehran, Iran; ^3^Statistical Research and Training Center, Tehran, Iran; ^4^Laboratory of Nutrition Research, National Nutrition and Food Technology Research Institute, Faculty of Nutrition Sciences and Food Technology, Shahid Beheshti University of Medical Sciences, Tehran, Iran

**Keywords:** malnutrition, prevalence, stunting, underweight, wasting, obesity

## Abstract

**Background and aim:**

Malnutrition is a major public health problem, especially in developing countries. The aim of this study was to analyze the trend in malnutrition among children under 5 years of age in Iran over recent decades and to estimate malnutrition status for 2020.

**Methods:**

This study took the form of a secondary analysis of the reports and data from three cross-sectional national surveys on children's nutritional status conducted between 1998 and 2017. Anthropometric indices, including markers of underweight, wasting, stunting, overweight, and obesity, were used as indicators of the nutritional status of children under 5 years. Malnutrition indicators are reported separately based on regional food security status. Linear mixed-effects modeling was used to predict the status of malnutrition indicators for 2020.

**Results:**

The results of this study indicated a downward trend in the prevalence of stunting, underweight, and wasting, from 15.4 to 4.8%, 10.9 to 4.3%, and 4.9 to 4.3%, respectively, between 1998 and 2017. The proportion of children at risk of overweight and prevalence of childhood overweight/obesity showed a downward trend between 2010 and 2017, from 3.73 to 3.02% and from 12.1 to 10.3%, respectively. However, the trend varied between different provinces. Estimates of the prevalence of malnutrition in 2020 also indicated a decrease in the prevalence of all indicators among children.

**Conclusion:**

Despite the decreasing trend in malnutrition over the past three decades, the prevalence of stunting, underweight, and wasting is still high in food-insecure provinces. Moreover, following the COVID-19 pandemic and its economic consequences, an increase in the prevalence of malnutrition, especially in food-insecure provinces, is plausible.

## Introduction

Nutritional status is the best worldwide indicator of children's wellbeing. Not only is growth assessment a tool for evaluating a child's health and nutritional status, but it also shows inequities in social development (1). Proper nutrition and health in the early years of life are among the indicators of sustainable development, and reducing child malnutrition by 2025 is one of the global sustainable development goals (SDGs) (2, 3).

Malnutrition is a broad term encompassing undernutrition (i.e., underweight, wasting, and stunting) on the one hand and over nutrition (including overweight and obesity) on the other. Malnutrition is a major public health problem, especially in developing countries. Both undernutrition and over nutrition have adverse health effects during all life stages (4). Undernutrition is the manifestation of insufficient access to food, disease, unsuitable feeding of children, limited access to health care services, or any possible combination of these factors. Certainly, the growth and development of children depend on various factors. Some of these factors, such as nutritional status and disease, have direct effects, while other factors, such as poverty and parents' illiteracy, are indirectly related to a child's growth and development through their contribution to poor food intake and disease (5, 6). The increasing global prevalence of obesity and its socioeconomic burden is currently a major public health problem (7, 8). Childhood overweight and obesity are associated with an increased risk of adverse health consequences, including such non-communicable diseases as diabetes, hypertension, and cardiovascular diseases (9).

Ensuring the health of the next generation by improving the nutritional status of infants and children is an important item on the public health agenda and a key challenge for the public healthcare system of any country (10). Determination of nutritional status, trend analysis, and forecasting of health indicators, especially in vulnerable groups (notably children), using data from national surveys, are crucial in policymaking and also in the planning of appropriate interventions (11).

According to national surveys conducted in Iran, the prevalence of child undernutrition (underweight and stunting) is decreasing, and that of obesity and overweight is increasing, which can be attributed to the rapid nutritional transition, urbanization, and changing food consumption patterns. However, the prevalence of malnutrition in some provinces of Iran is still much higher than the national average (12). Due to the coronavirus disease 2019 (COVID-19) pandemic and its effects on food security and access to health facilities, especially in vulnerable groups, it is likely that the nutritional status of young children has been affected (13). According to international reports, the COVID-19 pandemic as a health crisis has threatened the food security of millions of people around the world (14, 15). Although it is currently not possible to accurately predict the effects of this crisis, negative consequences can be expected in vulnerable groups, such as children and pregnant and lactating mothers, especially those who were food insecure before the pandemic (16).

In Iran, several national surveys on the prevalence of child malnutrition have been conducted. Despite the importance of trend analysis and forecasting of health indicators, including the nutritional status of children, there have been a very limited number of studies on the subject in Iran to date (12, 17). Although child malnutrition has been decreasing during recent decades, it is still necessary to evaluate the trend and extrapolate it into the future in order to determine the health status of the country and evaluate the achievements relative to SDG Target 2.2, which focuses on ending all forms of malnutrition, including stunting, wasting, and overweight, among children under 5 years of age (3). This study was therefore undertaken to analyze the trend in the prevalence of malnutrition (both under- and over-nutrition) among Iranian children under 5 years of age using data obtained from national surveys conducted during 1998–2017. Using these data, we also estimated the prevalence of child malnutrition in 2020.

## Materials and methods

This study was a secondary analysis of cross-sectional national surveys on child malnutrition conducted between 1998 and 2017. Anthropometric indices of underweight, wasting, stunting, overweight, and obesity were used as indicators of the nutritional status of children under 5 years of age. A weight-for-age z-score (WAZ), height/length-for-age z-score (HAZ/LAZ), or weight-for-height/length z-score (WHZ/WLZ) below −2 was considered to represent underweight, stunting, and wasting, respectively. Additionally, +2 ≥ WHZ/WLZ > +1 and WHZ > 2 were considered to represent risk of overweight and overweight/obesity, respectively (18).

### Statistical population and data collection

The statistical population consisted of all national surveys conducted during the last three decades that contained data on the anthropometric status of children under 5 years. Economic factors affecting the nutritional status of children were extracted from the Household Income and Expenditure Survey (HIES) (19). The National Institute of Health Research, the Ministry of Health and Medical Education, and the National Nutrition and Food Technology Research Institute provided information on national surveys. Additionally, food security status was determined based on a report by the National Food and Nutrition Security Monitoring System (SAMPAT) (20). By searching for national survey reports and interviewing key informants at the Ministry of Health, we identified five national surveys that contained anthropometric data on children under 5 years of age. Data from only three of these national reports were used in the current study: the first Anthropometric Nutrition Indicators Survey (ANIS-I), conducted in 1998 (21); the Iran Multiple Indicator Demographic and Health Survey (Ir.MIDHS), conducted in 2010 (22); and the Children Anthropometry, Nutrition, Development, Service indicators, Iran-1396 (CANDS-IR96), conducted in 2017 (23). The other two surveys, which were conducted in 1995 (the first national anthropometric survey of children) and 2004 [the second Anthropometric Nutrition Indicators Survey (ANIS-II)], were excluded due to unavailability of the final reports and incomplete or unavailable data. The only evidence that was obtained from these surveys was an article published in Persian that contained information on the prevalence of malnutrition among children under 5 years of age in general; the required information at the provincial level could not be extracted from this article (17).

The provinces of the country were divided into three categories according to food security status: secure, moderately insecure, and severely insecure (20). Anthropometric indicators are reported separately by food security status. Using the HIES data, we defined the economic–nutritional index (ENI) as the ratio of monthly household expenditure on meat, dairy products, fruits/vegetables, and cereals/grains to monthly expenditure on each of these groups in the optimal food basket.

Following the collection of data from national survey reports on the nutritional status of children under 5 years of age in Iran and on household expenditure on food, the trend in the nutritional status of children over the last three decades was analyzed. Based on the data obtained from the national reports, all concerns relating to the validity of data were considered when the data were gathered and analyzed. We used raw data on weight for height, weight for age, and height for age from the NCHS/1977 (National Center for Health Statistics/1977) in ANIS-I and the WHO/2006 (World Health Organization/2006) to evaluate malnutrition status. As information on overweight and obesity in children was not reported in the Ir.MIDHS, we used the data to calculate the prevalence of overweight and obesity based on weight for length/height Z-scores using the WHO child growth standards. All the aforementioned national surveys were financially supported by the Iran Ministry of Health.

### Statistical analyses

The prevalence of stunting, underweight, wasting, obesity, and overweight, as well as the number of children studied, were extracted from the reports. Due to a lack of data on the proportion of overweight and obese children in the 2010 survey, the indicators were calculated using raw data obtained from the Health Research Institute and the online version of the WHO Anthro Survey Analyzer software, after excluding outliers (+5 < Weight-for-Length/Height < −5).

In order to evaluate nutritional status, data on exclusive breastfeeding (EBF) up to 6 months of age that were available from the relevant national reports were examined, along with ENI. Food expenditure (not total household expenditure) was the only form of expenditure considered in this study. Given that household food expenditure is a proxy for household (and not individual) food consumption, and given the effects of household size and the gender and age of members of the household on food expenditure, adult male equivalent (AME) (24) was used to control for these relationships. For the calculation of ENI, two major variables (mean monthly consumption based on AME and weighted price) were calculated and extracted for each province in the studied year. Subsequently, ENI was calculated by dividing household expenditure on each food group by the cost of that food group in the optimal food basket. These analyses were conducted using the R Studio software package.

Linear mixed-effects models were used to determine the trends in anthropometric indices during recent decades and to estimate stunting, underweight, and wasting status for 2020; this is the method recommended by the World Health Organization (WHO). Estimation of the prevalence of overweight/obesity was not possible due to a lack of sufficient data on the relevant indicators in national surveys. Dependent variables were defined as the logit of HAZ, WHZ, and WAZ. The basic model included province and study year; the interaction between year and province was additionally included as a fixed effect. This type of model falls under the category of multilevel models, which are obtained by calculation of the restricted maximum likelihood. The full model was used to estimate the prevalence of malnutrition between 2000 and 2020: prevalence and the associated confidence interval (CI) were estimated using back-transformation. Linear mixed-effects modeling was also employed to construct a model accounting for food security. In this model, food security category (food-secure, moderately insecure, or severely food insecure) and year of study were included as fixed effects and province was included as a random effect. The total number of malnourished children was calculated by multiplying the prevalence estimate (with CI) by the population of children under 5 years. Statistical analyses were performed using the STATA 16 software package.

## Results

The results of the present study indicated a downward trend in the prevalence of stunting, underweight, and wasting, from 15.4 to 3%, 10.9 to 2.1%, and 4.9 to 3.1%, respectively. The proportion of children at risk of overweight and overweight/obese also showed a downward trend between 2010 and 2017, from 3.73 to 3.02% and from 12.1 to 10.3%, respectively.

Using the available data and linear mixed-effects models, the status of malnutrition in 2020 was predicted (Table 1). Model 1 is the crude model; in model 2, estimates were adjusted for ENI, province, and EBF in the first 6 months of life.

**Table 1:** Estimates of the prevalence of malnutrition in terms of number and proportion (with 95% confidence intervals) of malnourished children under 5 years of age in 2020 by regional food security.

		**Food secure**	**Moderately food insecure**	**Severely food insecure**	**Overall**
**Stunting (HAZ** < −**2)**					
Model 1	N	66,100	96,822	93,448	252,596
		(84,127–510,77)	(76,790–120,193)	(70,605–120,444)	(202,077–303,116)
	%	2.2	2.9	4.5	3
95% CI	(1.2–7.8)	(2.3–3.6)	(3.5–4.8)	(2.3–4.6)
Model 2	N	65,743	97,864	93,899	246,805
		(51,450–86,376)	(80,552–191,388)	(67,065–119,040)	(203,146–299,505)
	%	2.2	2.9	4.52	2.9
95% CI	(1.7–2.8)	(2.4–5.7)	(3.2–5.7)	(2.5–3.5)
**Underweight (WAZ** < −**2)**					
Model 1	N	51,979	61,098	67,490	176,817
		(37,256–72,410)	(46,074–80,463)	(47,762–94,902)	(143,138–218,917)
	%	1.73	1.83	3.25	2.1
95% CI	(1.24–2.41)	(1.38–2.41)	(2.3–4.57)	(1.7–2.6)
Model 2	N	54,040	60,050	60,870	167,053
		(36,440–65,743)	(49,326–80,552)	(41,200–89,517)	(151,441–223,943)
	%	1.8	1.8	2.9	2
95% CI	(1.2–2.2)	(1.5–2.4)	(2 −4.3)	(1.8–2.6)
**Wasting (WHZ** < −**2)**					
Model 1	N	99,150	86,806	85,142	261,016
		(73,311–132,501)	(66,774–110,177)	(62,299–114,214)	(210,497–319,955)
	%	3.3	2.6	4.1	3.1
95% CI	(2.44–4.41)	(2–3.3)	(3–5.5)	(2.5–3.8)
Model 2	N	97,033	88,799	81,332	271,923
		(72,490–129,517)	(66,240–107,825)	(60,870–119,040)	(223,943–362,957)
	%	3.2	2.6	3.9	3.2
95% CI	(2.4–4.3)	(1.3–9.2)	(2.9–5.7)	(2.6–4.3)

Model 1: raw model. Model 2: adjusted for economic–nutritional variables, province, and exclusive breastfeeding.

### Stunting

**Figure 1:**
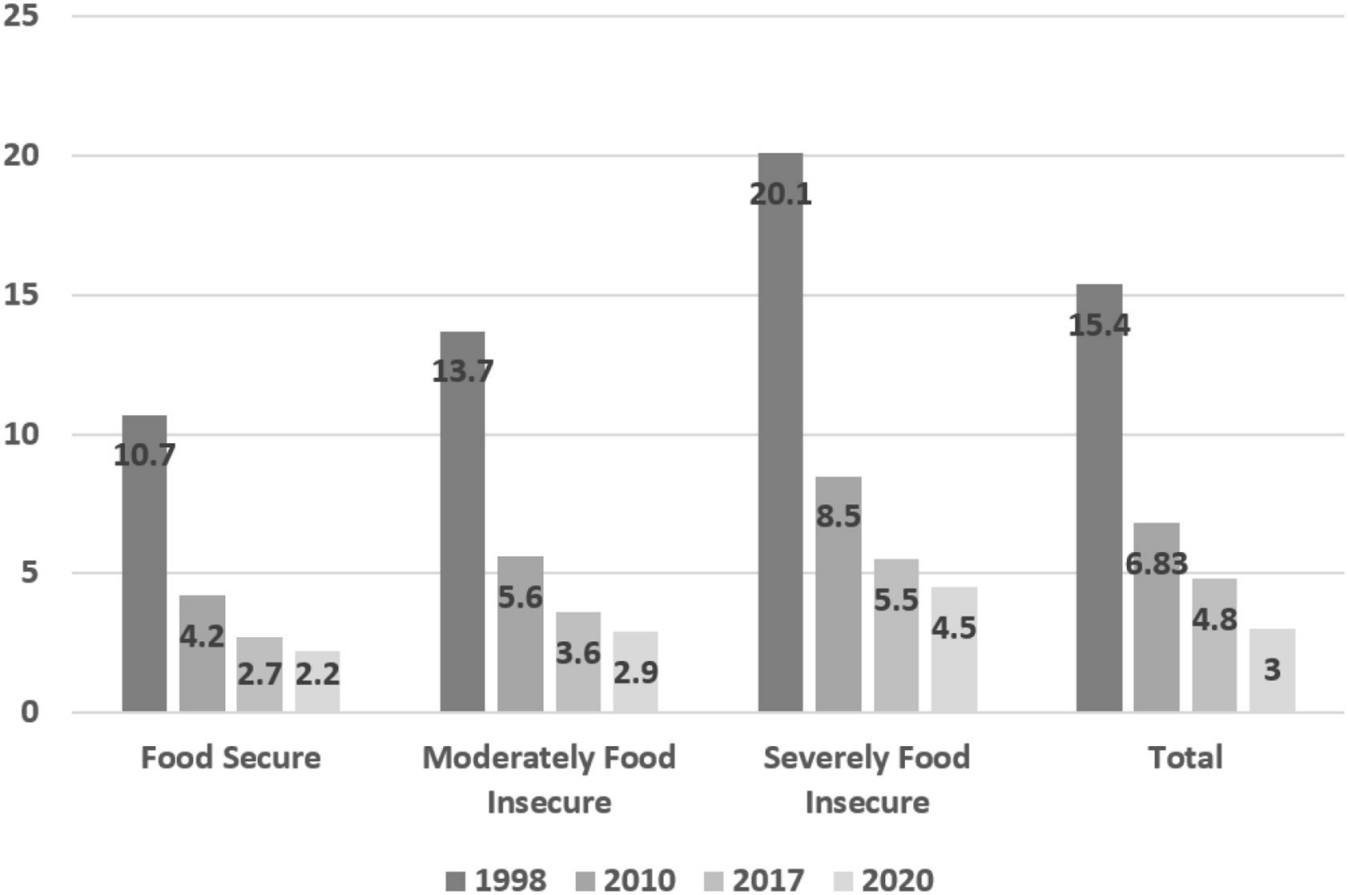
Prevalence of stunting (HAZ < −2) and 2020 estimate of prevalence in children under 5 years across the entire country and by provincial food security status.

There was a decreasing trend in the prevalence of nutritional stunting during the last three decades (Figure 1). Accordingly, the prevalence of stunting in 2020 was estimated at 2.2% in secure providences, 2.9% in moderately insecure provinces, and 4.5% in insecure provinces. After adjusting for economic variables, province, and EBF, these estimates of the prevalence of stunting did not change.

### Underweight

**Figure 2:**
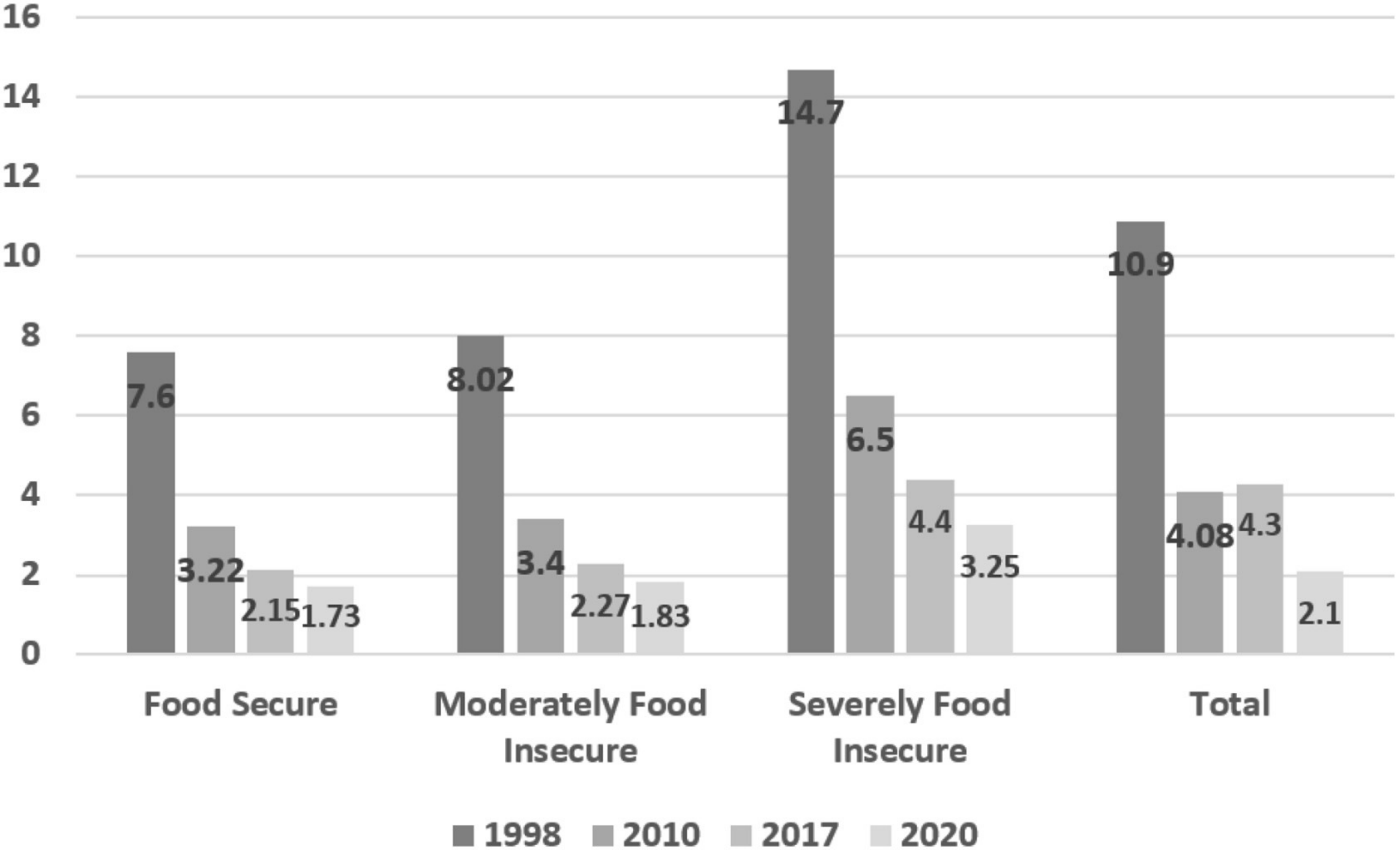
Prevalence of underweight (WAZ < −2) and 2020 estimate of prevalence in children under 5 years across the entire country and by provincial food security status.

Based on the results of surveys conducted over the last three decades, the prevalence of underweight among children aged under 5 years decreased from 10.9% in 1998 to 4.3% in 2017, which represented a slight increase in 2017 as compared with 2010 (4.08%). Nevertheless, the estimates of undernutrition in 2020 also showed the continuation of the decreasing trend across the entire country. Analysis of the prevalence of underweight according to food security status also indicated a decreasing trend (Figure 2).

### Wasting

**Figure 3:**
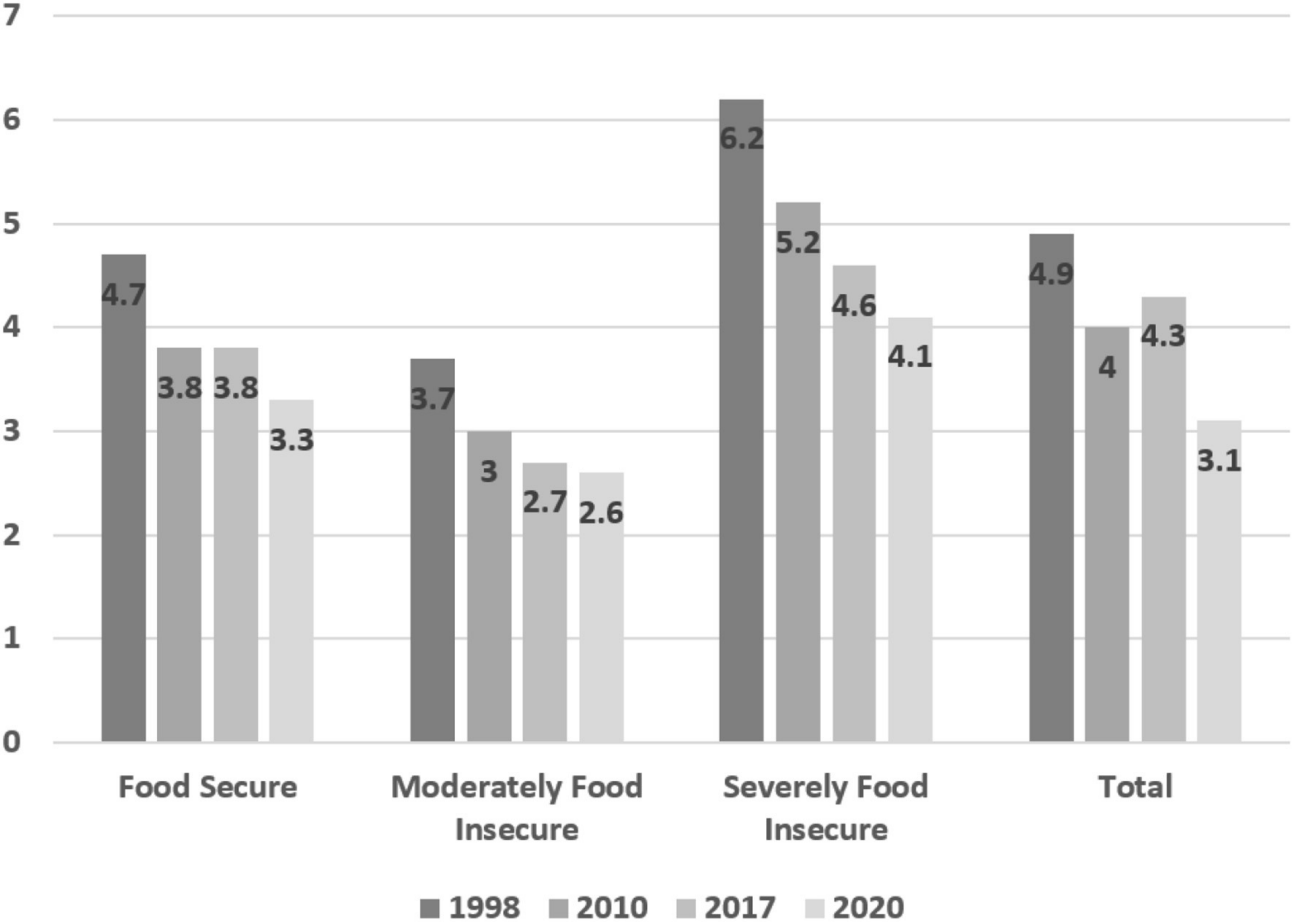
Prevalence of wasting (WHZ < −2) and 2020 estimate of prevalence in children under 5 years across the entire country and by provincial food security status.

The prevalence of wasting in Iran decreased from 4.9% in 1998 to 4.3% in 2017, although 2017 showed a slight increase compared with 2010 (4%). Estimates of the prevalence of wasting among children in 2020 also indicated a decrease. Analysis of the prevalence of wasting according to food security status also indicated a decreasing trend, except in the food-secure category, where the prevalence of wasting was constant between 2010 and 2017 (Figure 3).

### Overweight and obesity

**Figure 4:**
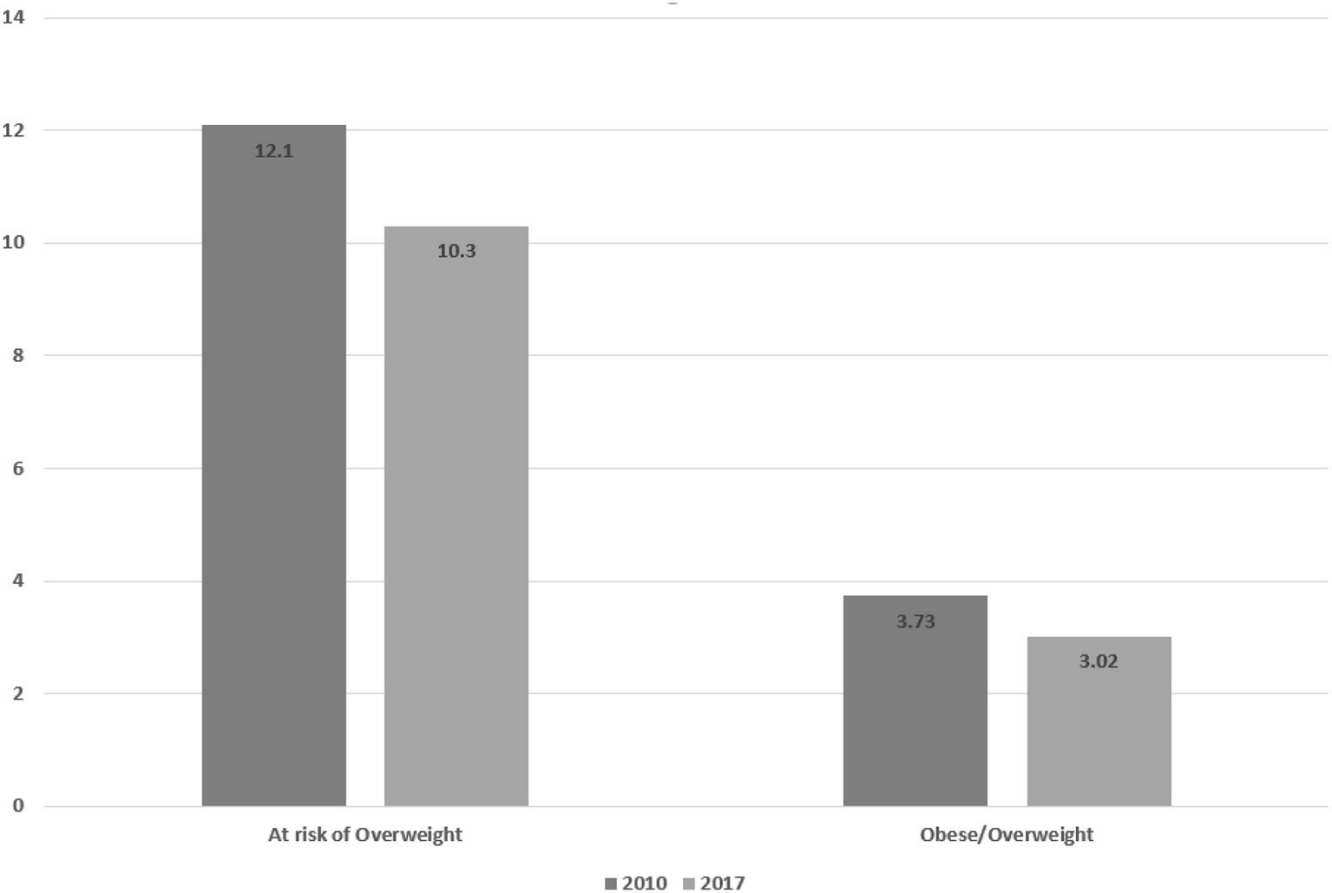
Prevalence of overweight/obesity and risk of overweight in children under 5 years.

As shown in Figure 4, the percentage of children at risk of being overweight and of overweight/obese children in 2017 had decreased relative to the percentages in 2010.

## Discussion

This study explored the trends in malnutrition in children under the age of 5 years in Iran using national survey results collected during 1998–2017; forecasts were also made for the pattern of malnutrition in this population of children for 2020. According to the results, there is a decreasing trend in the prevalence of stunting, underweight, and wasting in Iran.

The latest report of the UNICEF, WHO, and World Bank on global trends in malnutrition between 2000 and 2019 indicates a decrease in the occurrence of underweight and stunting in children under 5 years of age. Nevertheless, childhood malnutrition is still a crucial health concern in the world, making sustainable development goals difficult to achieve by the year 2030 for several countries (25).

Our study revealed a significant decrease in the prevalence of all forms of child undernutrition, including stunting, underweight, and wasting. Although the survey conducted in 2017 indicated a slight increase in the prevalence of underweight and wasting across the entire country compared with 2010, estimates of the prevalence of these forms of malnutrition in 2020 also showed the continuation of this downward trend. This trend was estimated to be similar in secure, moderately insecure, and insecure provinces. However, the slope representing the reduction in prevalence between the three investigated time points differed among various provinces; estimates of the reduction ranged between 50 and 85%. The highest estimated percentage reduction was for the prevalence of underweight across the whole country. Despite the decreasing trend estimated for the prevalence of stunting, underweight, and wasting, estimated rates were still high in severely food-insecure provinces. The reasons for the decreasing trend in undernutrition among Iranian children are likely to be rooted in changes in the determinants of child malnutrition. Although several studies have examined these determinants and there may be some differences from one country to another (26–32), the most common predictors are maternal education, the socioeconomic situation of the household, and maternal nutritional status (33).

Although women's education level has been found to be associated with certain health outcomes, including maternal mortality (34), health literacy (HL) is specifically a more important determinant of health and nutritional wellbeing (35, 36). A nationwide study in Iran has reported a low level of HL among Iranian adults (37), and a recent meta-analysis has revealed a marginal level of HL among Iranian women, showing a statistically significant association with self-efficacy and self-care behaviors (38).

Maternal nutritional status is an important determinant of child malnutrition (39). It is likely that the improvements in the nutritional status of pregnant mothers that occurred during the period 1998–2012, as reported in the latest National Integrated Micronutrient Survey (NIMS II), contributed to the decreasing trend in malnutrition in the country (40). Nevertheless, we do not have any recent data on maternal nutritional status, which warrants further national investigation.

It is noteworthy that there are now several policies and programs implemented in Iran that can influence the trend in child malnutrition. The most important of these relate to education for girls and women, child growth monitoring, oral rehydration, promotion of breastfeeding, dietary supplementation, nutrition for children under 5 years of age, immunization, and family planning (41).

One of the other determinants of child malnutrition is access to health facilities, clean water, and sanitation (29, 32, 42). Rapid unsustainable urbanization in Iran over the last 40 years, although it has resulted in several environmental and ecological challenges, may have concomitantly brought about greater access to health facilities for those households that previously resided in deprived rural areas (43). Furthermore, this form of rural–urban migration can also be accompanied by changes in resources, structures (including social, market, legal, and political systems), and values of the immigrant households, all of which can affect the nutritional status of children (27).

The incidence of overweight among children is also a global concern. In this study, the prevalence of overweight and obesity in children was calculated for the first time using the data from the 2010 Ir.MIDHS. The average prevalence of overweight across the entire country decreased between 2010 and 2017, with this trend covering all but a few provinces. The percentage of children at risk of overweight also decreased in 2017 compared with 2010 across the entire country and in most provinces. As obesity is influenced by various economic, biological, behavioral, and environmental factors, identification of its determinants can be important in policymaking and in selection of the appropriate approach to control it. Lifestyle changes and regular physical activity have been indicated to be the most important strategies for control of childhood obesity. In addition, there is a need to screen high-risk groups and implement intervention programs in developing countries (44).

Although the findings of this study on trends in the nutritional status of Iranian children under 5 years of age seem promising, it should be noted that the COVID-19 epidemic induced considerable changes in the dietary habits and lifestyle of the general population. Reports from a nationwide study (45) have revealed remarkable changes in various aspects of household dietary intake, including cereals, animal proteins (46), fast food consumption (47), intake of dairy products (48), and use of dietary supplements (49). Whether these changes will persist and/or will influence the nutritional status of children under 5 years of age remain to be established through future studies.

The major limitation of this study was the lack of access to data from all national surveys. Although the prevalence of malnutrition indicators was predicted for 2020 based on the results of past national surveys, the lack of data made it impossible to investigate the impact of the COVID-19 pandemic on the nutritional status of children. In Iran, no national survey has been conducted on the determinants of overweight and obesity in children, and there are few studies on childhood obesity compared with obesity among adults. Furthermore, because national data were available only for two time points, it was not possible to predict the prevalence of overweight and obesity in 2020. The other limitation of this study was the criteria employed for assessment of malnutrition in ANIS-1. Based on the existing report and existing criteria for the definition of malnutrition at that time, NCHS/WHO/CDC growth charts were used for the definition of malnutrition among children.

Based on the findings of this study, it can be suggested that determinants of different forms of child malnutrition (both under- and over-nutrition) should be investigated and identified regionally in those provinces where the prevalence rates are high and/or the prevalence trend is increasing. In addition, in terms of planning to control and prevent various types of malnutrition, in addition to national goals for achieving sustainable development, programs should be planned, designed, and implemented at the regional and provincial levels. These programs should consider the local determinants of malnutrition and overweight, including lack of knowledge regarding feeding practices among mothers, feeding of children with foods with low nutritional value and nutrient density, inappropriate forms of child care, short periods of exclusive breastfeeding, lack of access to health services, and household food insecurity. Nutrition-sensitive interventions should be planned, executed, and evaluated with the purpose of poverty reduction and household empowerment through emphasis on interventions affecting child malnutrition. Furthermore, for the purpose of strategic planning and evaluation of ongoing progress, data on the status of children's growth should be collected, analyzed, and interpreted continually and regularly through extension of the food and nutrition surveillance system to all provinces of the country and regular reports should be presented to policymakers. The current economic situation and the conditions caused by the COVID-19 pandemic must be taken into account in the planning of interventions and prioritization of particular areas.

## Conclusion

Despite the decreasing trend in malnutrition over the past three decades, the prevalence of stunting, underweight, and wasting is still higher than the national level in food-insecure provinces. Moreover, with the COVID-19 pandemic and the increased risk of food insecurity, especially due to the global economic recession, it is expected that the prevalence of malnutrition in children will increase. Forecasting and modeling of the future prevalence of malnutrition can facilitate the identification of areas at high risk, emphasizing the need to design and implement effective interventions at the local level. Furthermore, analysis of trends in malnutrition status and prediction of future trends could indicate whether the target for child malnutrition under SDG-2.2 is achievable by 2025.

## Data availability statement

The data analyzed in this study is subject to the following licenses/restrictions: Datasets are in Persian and are available from the corresponding author upon reasonable request. Requests to access these datasets should be directed to nikooyeh11024@yahoo.com.

## Author contributions

DG, BN, TN, AF, and HE-Z conceived and designed the study. ZY wrote the first draft of the manuscript. HR contributed to data preparation. RR, DG, and BN contributed to data analysis. All authors have read, commented on, and agreed to the final version of the manuscript.
